# Serum protein signature of coronary artery disease in type 2 diabetes mellitus

**DOI:** 10.1186/s12967-018-1755-5

**Published:** 2019-01-24

**Authors:** Ramu Adela, Podduturu Naveen Chander Reddy, Tarini Shankar Ghosh, Suruchi Aggarwal, Amit Kumar Yadav, Bhabatosh Das, Sanjay K. Banerjee

**Affiliations:** 1 Drug Discovery Research Center, Translational Health Science and Technology Institute (THSTI), Faridabad, Haryana 121001 India; 2 Mediciti Institute of Medical Sciences, Medchal Mandal, Ranga Reddy Dist., Ghanpur, Telangana India; 3 Centre for Human Microbial Ecology, Translational Health Science and Technology Institute, Faridabad, 121001 India; 4 Present Address: Department of Pharmacy Practice, National Institute of Pharmaceutical Education and Research, NIPER, Guwahati, Assam India

**Keywords:** Type 2 diabetes mellitus, Coronary artery diseases, Cytokines/chemokines, Apolipoproteins, Adipokines, Metabolic hormones and biomarkers

## Abstract

**Background:**

Coronary artery disease (CAD) is the leading cause of morbidity and mortality in patients with type 2 diabetes mellitus (T2DM). The purpose of the present study was to discriminate the Indian CAD patients with or without T2DM by using multiple pathophysiological biomarkers.

**Methods:**

Using sensitive multiplex protein assays, we assessed 46 protein markers including cytokines/chemokines, metabolic hormones, adipokines and apolipoproteins for evaluating different pathophysiological conditions of control, T2DM, CAD and T2DM with CAD patients (T2DM_CAD). Network analysis was performed to create protein-protein interaction networks by using significantly (p < 0.05) altered protein markers in each disease using STRING 10.5 database. We used two supervised analysis methods i.e., between class analysis (BCA) and principal component analysis (PCA) to reveals distinct biomarkers profiles. Further, random forest classification (RF) was used to classify the diseases by the panel of markers.

**Results:**

Our two supervised analysis methods BCA and PCA revealed a distinct biomarker profiles and high degree of variability in the marker profiles for T2DM_CAD and CAD. Thereafter, the present study identified multiple potential biomarkers to differentiate T2DM, CAD, and T2DM_CAD patients based on their relative abundance in serum. RF classified T2DM based on the abundance patterns of nine markers i.e., IL-1β, GM-CSF, glucagon, PAI-I, rantes, IP-10, resistin, GIP and Apo-B; CAD by 14 markers i.e., resistin, PDGF-BB, PAI-1, lipocalin-2, leptin, IL-13, eotaxin, GM-CSF, Apo-E, ghrelin, adipsin, GIP, Apo-CII and IP-10; and T2DM _CAD by 12 markers i.e., insulin, resistin, PAI-1, adiponectin, lipocalin-2, GM-CSF, adipsin, leptin, Apo-AII, rantes, IL-6 and ghrelin with respect to the control subjects. Using network analysis, we have identified several cellular network proteins like PTPN1, AKT1, INSR, LEPR, IRS1, IRS2, IL1R2, IL6R, PCSK9 and MYD88, which are responsible for regulating inflammation, insulin resistance, and atherosclerosis.

**Conclusion:**

We have identified three distinct sets of serum markers for diabetes, CAD and diabetes associated with CAD in Indian patients using nonparametric-based machine learning approach. These multiple marker classifiers may be useful for monitoring progression from a healthy person to T2DM and T2DM to T2DM_CAD. However, these findings need to be further confirmed in the future studies with large number of samples.

**Electronic supplementary material:**

The online version of this article (10.1186/s12967-018-1755-5) contains supplementary material, which is available to authorized users.

## Background

Type 2 diabetes mellitus (T2DM) is a chronic metabolic disease associated with higher blood glucose levels as a result of insufficient insulin secretion, insulin action or both. T2DM is influenced by both host genetics and environmental factors including age, family history, diet and sedentary life style. The global burden of T2DM has been estimated at 425 million and it is going to increase 48% i.e., 625 million by the year 2045 [1]. T2DM is a major risk factor for developing micro and macro vascular complications [2]. Patients with T2DM have two to three-fold higher cardiovascular risk than non-diabetic subjects [1]. Persistently high blood glucose levels may cause vascular damage and develop vascular complications like coronary artery disease (CAD), which further lead to angina or myocardial infarction. Diabetic patients were unaware of the cardiovascular complications until they were hospitalized with angina or myocardial infarction. Early prediction or detection of the disease may prevent the disease progression by therapeutic intervention and management plan. Therefore, there is a need to find specific markers for the detection of different levels of diseases severity or progression of T2DM associated with CAD. Until now, most of the studies reported single or very few numbers of markers to understand the disease progression [3] and limited number of studies used multiple markers approaches giving consideration of different pathophysiological process, like lipoprotein metabolism, hormonal imbalance and inflammation [4]. Several prospective and randomized studies such as United Kingdom Prospective Diabetes Study (UKPDS), Action to Control Cardiovascular Risk in Diabetes (ACCORD), Action in Diabetes and Vascular Disease Preterax and Diamicron Modified Release Controlled Evaluation (ADVANCE) and Veterans Administration Diabetes Trial (VADT) show that regulation of glucose has little or no effect on cardiovascular complication and mortality [5]. Canakinumab Anti-inflammatory Thrombosis Outcome Study (CANTOS) with cankanemib anti-inflammatory therapy targeting IL-1beta shown reduced recurrent cardiovascular events and decreased IL-6 levels dose depended manner [6].

CANTOS study as well as previous literature demonstrated that inflammation plays pivotal role in the development and progression of many complex diseases such as hypertension, dyslipidaemia, diabetes, and cardiovascular diseases (CVD) [6, 7]. Data indicates that inflammation could be the bridging link between T2DM and CVD. Chronic inflammation works through different mechanisms like endothelial dysfunction, oxidative stress, and insulin resistance [8]. Among different cells, macrophages and adipocytes play important role to induce inflammation in diabetes. Cytokines are cell-signaling molecules in inflammatory state and secretes from macrophages [9]. Similar to macrophages, adipocytes are also very sensitive to inflammation and secrete different adipokines such as leptin, adiponectin, resistin, and pro-inflammatory factors like TNF-α, IL-1β, IL-6, and PAI-1. Retained lipoproteins with other atherogenic factors that observed during atherosclerosis process, activates endothelial cells to recruit more immune cells like monocytes, which further differentiate into macrophages and activate other inflammatory signaling pathways [10]. In the process of atherosclerosis development, the expression of lipoprotein surface molecules such as apolipoproteins i.e., atherogenic (Apo-B) and anti-atherogenic (Apo-AI) molecules [11] may alter and cause coronary artery diseases [12]. We hypothesised that different cells like macrophages, adipocytes, and endothelial cells were communicating through metabolic hormones, cytokine/chemokine, adipokine and apolipoproteins to regulate insulin sensitivity, lipid metabolism, and inflammation. Perturbation in signaling molecules homeostasis due to environmental or genetic changes may contribute in T2DM and CAD progression. Therefore, a set of markers perturbed at different stages of the disease progression, may be potential biomarkers for predicting diseases phenotypes. To identify potential biomarkers of T2DM with or without cardiovascular complication, different omics approaches targeting multiple proteins, metabolites, microRNAs and long non-coding RNAs are preferred [13]. In the present study, we measured protein markers in patients with T2DM, CAD, T2DM_CAD and healthy controls to identify a disease-specific panel through non-parametric based machine learning classification to distinct different stages of disease progression.

## Methods

### Patient selection and consent process

Total 127 subjects, including male and female (90 male and 37 female) aged 35–65 years [median 44 (Inter quartile range (38–52)], were randomly recruited from Mediciti Hospital, Hyderabad. The study conforms to the principles outlined in the Declaration of Helsinki and was approved by the Mediciti Ethics Committee (Institutional human ethics committee), Hyderabad. All patients given detailed information about the study and they have provided written consent before enrolling into the study. Four different groups of subjects enrolled into the study.

### Inclusion and exclusion criteria for selection of study subjects

*Group 1*: Control (CT, n = 26) subjects had no prior history of T2DM, hypertension, coronary artery diseases or any other cardiovascular diseases, and were not taking medication for any chronic medical condition. Fasting blood glucose, HbA1c and blood chemistry were normal. *Group 2*: Type 2 diabetes (T2DM, n = 53) subjects with HbA1c levels ≥ 6.5% as per American Diabetes Association (ADA) guidelines with proven history of T2DM and no other complications. *Group 3*: Coronary artery diseases (CAD, n = 21) subjects were diagnosed based on positive medical history (myocardial infarction, angina pectoris and coronary artery bypass graft) and/or ischemic changes on a conventional 12-lead ECG, which included ST-segment depression or Q-wave changes [14]. Coronary artery disease subjects were identified in inpatient setting of cardiac catheterization unit in Mediciti hospital by the cardiologist. This group had no prior history of T2DM. *Group 4*: Type 2 diabetes with coronary artery diseases (T2DM_CAD, n = 27) subjects were coronary artery disease as defined for group 3 but patient had HbA1c levels ≥ 6.5% and prior history of T2DM. Information regarding demographic, clinical, and angiographic data was also collected from all patients. Fasting samples were collected from the patients prior to the percutaneous coronary intervention (PCI) or coronary artery bypass graft (CABG). Clinical or laboratory evidence of chronic diseases conditions like liver failure, renal failure (serum creatinine levels > 1.5 mg/dl), type 1 diabetes, cancer, thyroid disease and pregnancy subjects were excluded from the study.

### Measurement of circulatory protein markers

Serum concentration of cytokines/chemokines panel (IL-1β, IL-17, IL-1ra, basic FGF, IL-2, eotaxin, IL-4, G-CSF, IL-5, GM-CSF, IL-6, IFN-γ, IL-7, IP-10, IL-8, MCP-1(MCAF), IL-9, MIP-1α, IL-10, MIP-1β, IL-12(p70), PDGF-BB, IL-13, RANTES, IL-15, TNF-α, VEGF) and metabolic hormone panel (C-peptide, ghrelin, GIP, GLP-1, glucagon, insulin, leptin, PAI-1 (total), resistin, and visfatin) were measured using Bio-Plex Pro human cytokine Grp I panel 27-plex (Cat#M50-0KCAF0Y) and Bio-Plex Pro human diabetes panel 10-plex (Cat#171-A7001M), respectively. Adipokine panel (lipocolin-2, adiponectin, and adipsin) and apolipoproteins panel (Apo AII, Apo-AI, Apo -CII, Apo-CIII, Apo-B, Apo-E) were measured by using Milliplex MAP human adipokine magnetic bead panel 1 (Cat#HADK1MAG-61K) and Milliplex MAP human apolipoprotein magnetic bead panel (Cat#APOMAG-62K), respectively. On the day of experiment, frozen serum samples were thawed, mixed by vortexing, and then centrifuged at 10,000 rpm for 5 min to isolate debris. All experiments were performed according to the manufacturer’s instructions. Briefly, serum samples diluted with sample diluent 1:4 fold for the cytokine/chemokine and metabolic hormones analysis, and 1:4000 fold for adipokine and apolipoprotein analysis. The diluted serum samples (25 µl) were mixed with biomagnetic beads in a 96 well flat bottom plates and further analysed by using the Bio-Plex-200 system (Bio-Rad Corp. USA) All cytokines/chemokines, metabolic hormones, adipokines and apolipoproteins standards were provided by the manufacturers. Acquisition gates were set at 8000–15,000 and 50 events per bead were acquired. Mean fluorescence intensity was measured by using Bioplex manager software version 5.0 (Bio-Rad) and compared to a standard curve to generate concentration values [15]. Values below the range of the standard curve were set to the lower limit of detection.

### Statistical analysis, classification and visualization

The majority of statistical analysis and visualizations were performed using the various modules of the R programming interface. In order to obtain the fold change, we used the median values of each protein marker obtained across all the healthy controls (referred to as the ‘control median’). The fold change of a given marker for a given patient was then obtained as the log-ratio of the value of the marker in that patient divided by the control-median corresponding to that marker. The various modules used for this purpose were randomForest (Random forest classifications), dudi.pca of the ade4 package (for performing Principal Component Analysis), dunn.test (for performing post hoc dunn’s tests of pairwise comparisons across cohorts), and kruskal.test (for identifying the significantly different markers for different cohorts). Only p < 0.05 were considered as significant. We used two-stage approach to select the specific markers (Table 2). In the first step, we selected all those protein features that were significantly different between two groups at a nominal p-value < 0.05. Then, in the second step, on this subset, we applied a Benjamini–Hochberg correction and selected those features with corrected false discovery rate (fdr) p-value < 0.15. Correlations between markers, and between clinical parameters and protein markers were obtained using kendall’s tau (corr function of R) and spearman correlation was used to find the correlation. R value 0.3 was set as threshold and significance was considered as p < 0.05. The different modules for visualizations were heatmap.2 (for heatmap), Between Class Analysis is a specialized form of ‘supervised’ Principal Component Analysis (PCA), with respect to the instrumental variable (in this the class). It provided better resolution and provides a better analysis for marker identification as compared to the PCA factoextra (for PCA biplot showing the association of the markers with different patient marker profiles), ggcorrplot (for plotting the correlations as heatmaps) and s.class function of the ade4 package (for visualization of the class-based resolution of the patient marker profiles obtained using the PCA). In-house codes were written in Perl for computing the pair-wise variations among patient marker profiles using the J-divergence measure [16].

## Results

A total 127 subjects were randomly selected and enrolled in the study (Table 1). Clinical and biochemical characteristics were represented in the Table 1. Male and female subject’s ratio was not matching in the study groups as male subjects were more prone to CAD than females. Fasting blood glucose and glycated haemoglobin (HbA1c) levels were significantly (p < 0.001) increased in T2DM and T2DM_CAD groups as compared to control.

**Table 1:** Clinical and biochemical variables in study groups

Variables	Control (n = 26)	T2DM (n = 53)	CAD (n = 25)	T2DM_CAD (n = 26)
Age (years)	44.3 ± 9.4	46.5 ± 8.5	48.8 ± 6.6	51.2 ± 8.2
Gender (male/female)	15/11	28/25	24/1	23/3
BMI	25.30 (22.31–28.30)	26.5 (23.5–29.9)	25.0 (22.6–26.7)	25.3 (22.7–26.7)
Systolic BP (mmHg)	124.3 (121.0–130.6)	136.2 (127.5–149.6)	133.0 (123.8–139.0)	133.0 (112.7–145.4)
Diastolic BP (mmHg)	81.3 (77.6–84.0)	82.8 (78.2–93.0)	80.5 (74.7–85.7)	82.8 (73.4–89.3)
HbA1C (%)	5.4 (5.2–5.7)	7.9 (7.2–9.1)^a^	5.4 (5.2–5.6)	8.6 (7.8–9.4)^a, b^
FBS (mg/dl)	97.0 (88.0–107.0)	188.0 (150.0–239.2)^a^	97.5(83.7–109.7)	191.5(154.8–256.5)^a, b^
Creatinine (mg/dl)	0.8 (0.7–0.9)	0.9 (0.7–1.0)	0.9 (0.8–1.0)	1.0 (0.8–1.2)
eGFR (ml/min/1.73m^2^)	100 (85–117)	92.5 (80.2–109.0)	91.5 (81.2–105.2)	80.5 (66.7–99.0)
Uric acid (mg/dl)	4.2 (3.5–5.7)	4.0 (3.3–4.8)	4.6 (4.1–6.1)	4.3 (3.4–5.5)
CK-MB (mg/dl)	20 (16.0–24.0)	17.0 (13.7–22.0)	22.5 (19.5–26.7)	22.5 (20.0–29.5)
Smoking history (yes/no)	4/22	13/40	10/15	16/10
Alcoholic history (yes/no)	7/19	14/39	8/17	19/6
FRS (%)	0.9 (0.5–3.8)	1.5 (0.3–6.7)	5.2 (2.15–9.3)	5.3 (2.7–10.7)^a^
ASCVD (%)	1.9 (0.9–3.9)	4.0 (2.4–16.4)^a^	10 (3–15.1)^a^	15.4 (9.3–22.2)^a^
Diabetic duration (years)	–	2 (1–4)	–	2 (1–5)
Hypertension history	–	25	16	14
Patients were on hypertensive drugs	–	18	13	14
Patients were on anti-diabetes medication	–	34	–	17
Patients were on anti-platelet and statin therapy	–	–	25	26

Data are mean (SD) and median (Q1–Q3) for normally distributed and non-normally distributed variables respectively

*BMI* Body mass index, *FBS* fasting blood sugar, *HbA1c* glycated hemoglobin, *eGFR* estimated glomerular filtration rate, *FRS* Framingham Coronary Heart Disease Risk Score in 10 years, *ASCVD* estimate risk score for atherosclerotic cardiovascular disease in 10 years. Anti-platelet and Statin therapy was given to the all the patients for the prophylaxis for the CAD event

^a^p < 0.05 compared to control

^b^p < 0.05 compared to CAD

### Serum protein markers levels in study groups

**Fig. 1:**
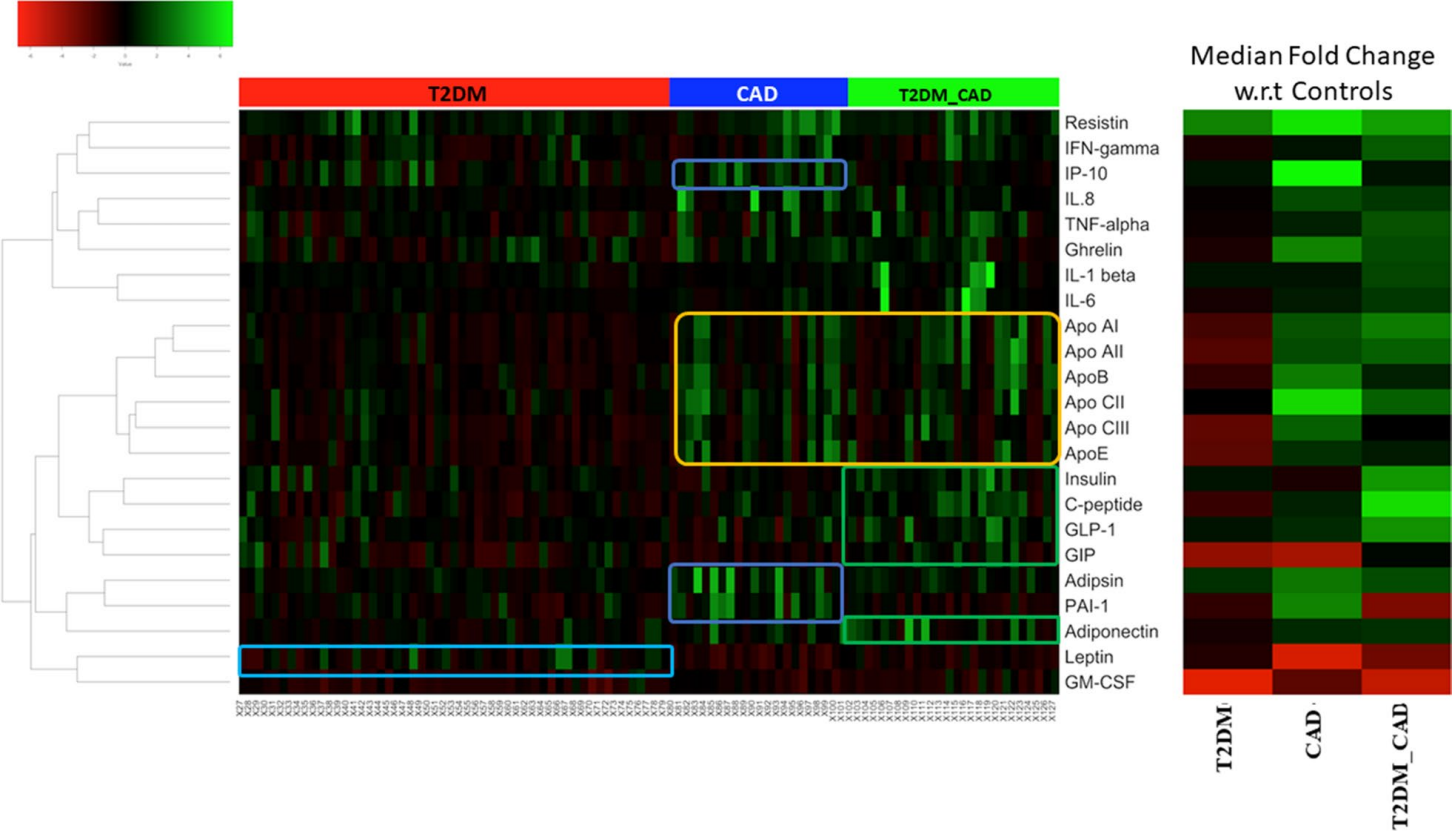
Heatmap showing the fold change of the various clinically significant markers across all the individuals belonging to the three different disease states. In order to obtain the fold change, the median values of each clinical marker was obtained across all the healthy controls (referred to as the ‘control median’). The fold change of a given marker for a given patient was then obtained as the log-ratio of the value of the marker in that patient divided by the control-median corresponding to that marker. Four distinct sets of correlated protein markers (CLs) are highlighted by dark blue, light blue, yellow and green boxes on heatmap. The median fold change in each disease cohort versus the control medians of each marker is also shown

Levels of 45 protein markers in the serum samples of enrolled subjects were measured. Four cytokines IL-2, IL-7, IL-15 and MIP-1α were excluded from out of 46 protein markers due to detection limits of the present assay. Fold change of the various clinically significant markers across all the individuals belonging to the three different disease states i.e., T2DM, CAD and T2DM_CAD represented in heat map. The median fold change in each disease cohort versus the control medians of each marker is also shown (Fig. 1).

### Serum protein marker levels in T2DM_CAD as compared with control, T2DM and CAD group

We observed alteration of individual serum protein markers levels in the study groups (Table 2). Metabolic hormones (i.e., C-peptide, GLP-1, and insulin), inflammatory markers (i.e., TNF-α, IL-1β, and eotaxin) and adipokines (i.e., resistin, adipsin and lipocalin-2) levels were significantly (p < 0.05; fdr corrected p-value < 0.15) increased in T2DM_CAD group, while levels of GM-CSF (p < 0.001; fdr corrected p-value < 0.12) and PAI-1 were significantly (p < 0.05; fdr corrected p-value < 0.12) decreased in T2DM_CAD as compared to the control subjects. Similarly, metabolic hormones i.e., GLP-1, GIP and insulin levels were significantly (fdr corrected p-value < 0.05) increased, and leptin levels were significantly decreased in T2DM_CAD group (fdr corrected p-value < 0.10) as compared with T2DM subjects. Inflammatory markers i.e., TNF-α and IL-6; adipokine such as adipsin and apolipoproteins i.e., Apo-AI, Apo-AII, and Apo-E levels were significantly (fdr corrected p-value < 0.15) increased in the T2DM_CAD group as compared to T2DM group.

**Table 2:** Serum cytokine/chemokine, metabolic hormones adipokines and apolipoproteins levels in study groups

Variables (characteristics at study visit)	Control (n = 26)	T2DM (n = 53)	CAD (n = 21)	T2DM_CAD (n = 27)
*Units (picogram/ml)*				
C.peptide	1911.5 (1053.7–3071.3)	1705.3 (1041.0–2078.9)	2172.2 (1718.0–2780.3)^b^**	2874.2 (1965.5–3647.3)^a^*^b^***^c^
Ghrelin	473.0 (346.8–662.8)	494.2 (339.4–676.0)	624.0 (562.4–809.3)^a^**,^b^**	588.5 (449.7–687.5)
GIP	642.4 (544.9–1005.2)	419.5 (212.1–824.9)^a^**	386.7 (280.6–556.6)^a^***	631.4 (534.4–933.6) ^b^*, ^c^***
GLP-1	170.7 (147.9–182.8)	173.9 (152.3–177.8)	177.0 (144.18–195.56)	194.4 (175.1–237.4)^a^*,^b^***,^c^**
Glucagon	201.4 (181.2–219.0)	169.25 (132.33–212.71)^a^**	178.02 (143.45–201.81)	202.04 (154.61–262.69)
Insulin	1092.4 (474.2–1493.1)	1206.1 (600.8–1620.4)	1003.4 (487.7–1275.0)	1660.9 (1274.7–2213.7)^a^**,^b^***,^c^***
Leptin	6918.8 (2888.0–10,986.4)	6532 (4215–9704)	2567 (1771–5338)^a^***,^b^***	5186 (3364–6006)^b^*
Resistin	4165 (3282–6456)	6610 (4447–7617)^a^**	8034 (7032–12,190)^a^***	7193 (6480–8299)^a^***
Visfatin	1741.2 (974.2–2162.1)	1786.8 (1155.0–2440.7)	2090.4 (1677.3–2479.1)	2088 (1484–3302)
IL.1b	1.9 (1.390–2.440)	2.1 (1.770–2.560)	2.1 (1.986–2.357)	2.4 (1.950–4.160)^a^*
IL.1ra	107.46 (52.42–141.12)	93.93 (48.51–155.91)	99.41 (43.98–120.18)	88.38 (46.15–150.37)
IL.4	10.55 (8.24–13.96)	11.18 (9.45–14.05)	11.30 (9.32–13.44)	10.9 (7.935–14.450)
IL.5	3.4 (1.500–5.497)	3.3 (1.860–4.770)	3.0 (1.365–4.735)	3.6 (1.998–4.985)
IL.6	11.4 (8.22–12.84)	10.0 (6.21–13.11)	13.4 (9.955–18.607)	14.6 (8.662–19.793)^b^***
IL.8	23.56 (13.94–37.74)	23.71 (13.33–33.07)	32.90 (20.59–114.59)	31.93 (20.39–41.76)
IL.9	10.8 (6.7–14.3)	11.7 (8.18–15.00)	10.6 (6.8–13.6)	11.2 (6.8–13.8)
IL.10	12.8 (7.5–19.3)	12.58 (6.08–15.84)	13.18 (11.89–16.58)	12.2 (8.082–14.399)
IL.12	42.77 (21.95–57.14)	45.54 (23.87–64.72)	47.52 (19.54–61.02)	44.97 (26.96–70.29)
IL.13	34.43 (20.34–47.57)	25.03 (15.29–41.08)	23.31 (11.34–34.86)	28.30 (16.23–51.08)
IL.17	44.84 (12.22–72.68)	41.96 (11.48–67.74)	29.1 (3.493–86.780)	43.09 (12.29–70.50)
Eotaxin	93.00 (71.26–113.32)	103.12 (81.47–131.97)	113.61 (93.62–136.47)^a^**	117.12 (93.48–137.64)^a^*
FGF-basic	10.7 (6.7–14.0)	7.8 (2.2–11.6)	10.0 (3.9–17.0)	10.4 (7.8–16.03)
G.CSF	33.34 (16.97–42.09)	28.25 (14.07–43.22)	32.56 (9.12–50.43)	28.65 (10.34–55.58)
GM.CSF	56.39 (43.78–71.12)	34.28 (31.98–42.17)^a^***	46.60 (38.05–53.55)^a^**^b^**	37.55 (31.76–47.39)^a^*
IFN.g	90.68 (36.40–121.04)	83.60 (41.74–105.83)	97.21 (51.86–127.01)	114.56 (80.46–148.43)
IP.10	903.2 (669.0–1064.8)	974.3 (659.3–1283.5)	1253.4 (909.5–1638.2)^a^*,^b^**	962.6 (768.0–1053.7)^c^***
MCP.1	53.53 (48.38–66.22)	48.41 (33.02–54.22)	50.42 (41.04–55.04)	48.12 (30.85–62.78)
PDGF.BB	5050.9 (1063.8–7555.0)	6681.0 (4669–8509)	6911.0 (6572–8468)^a^**	6595.2 (3097.8–8398.9)
MIP.1b	101.97 (41.72–135.60)	76.42 (53.49–159.02)	123.97 (73.31–190.44)	120.22 (71.64–139.01)
Rantes	15,219 (7372–18,183)	14,305 (12,526–15,954)	13,552 (11,212–16,808)	13,248 (11,908–17,131)
TNF.alpha	34.60 (27.94–41.50)	33.87 (21.26–44.26)	37.66 (28.55–53.59)	40.73 (33.78–63.81)^a^*,^b^**
VEGF	114.50 (54.65–188.68)	127.74 (65.32–181.43)	134.96 (51.93–225.07)	132.70 (72.67–207.03)
*Units (ng/ml)*				
Adiponectin	8975.2 (6466.4–9677.6)	8447.2 (5549.6–10,786.6)	10,033.5 (8561.3–14,092.3)	10,146.4 (5094.9–19,997.5) a
Adipsin	5809.8 (3823.6–6947.1)	6747.1 (4480.8–7179.4)	8070.2 (5327.3–15,827.5)^a^***,^b^**	7261.2 (6133.7–9968.0)^a^***,^b^**
Lipocalin-2	197.3 (155.27–259.00)	262.6 (187.87–344.5)^a^**	318.8 (249.7–395.3)^a^***,^b^ ^b^	294.2 (123.39–455.10)^a^
PAI.1	138.7 (114.2–146.4)	120.7 (82.2–162.0)	172.7 (132.9–329.9)^a^***,^b^***	96.9 (68.2–139.4)^a^*, ^c^***
Apo.AI	1405.5 (1068.6–1621.2)	1209.5 (895.8–1383.1)^a^	1604.6 (1231.9–2491.4)^b^***	1635.3 (1016.6–2385.6)^b^**
Apo.AII	307.2 (249.4–351.1)	266.5 (222.0–299.1)	360.0 (286.1–474.6)^b^***	352.7 (240.4–602.3)^b^*
Apo.B	120.4 (79.3–191.5)	109.3 (69.6–130.3)	156.8 (87.3–246.7)^b^**	136.3 (79.9–283.1)
Apo.CII	63.54 (47.87–82.56)	68.07 (43.92–81.36)	99.8 (73.05–141.21)^a^***, ^b^***	83.10 (37.45–114.26)
Apo.CIII	326.5 (218.8–483.4)	247.32 (157.2–319.7)	357.5 (247.5–564.8)^b^**	329.0 (149.4–381.1)
Apo.E	91.0 (65.5–105.0)	76.6 (59.1–86.7)	102.5 (75.4–176.1)^b^***	97.8 (49.9–124.0)^b^

Continuous variables are reported as median (25th–75th percentile) as not found normally distributed. Comparisons between outcome groups by the Kruskal–Wallis and Dunn’s tests

*FDR* false discovery rate

* p < 0.15, ** p < 0.1 and *** p < 0.05

^a^p < 0.05 as compared to control

^b^p < 0.05 as compared to T2DM

^c^p < 0.05 as compared to CAD

In the present study, we also compared protein markers alteration in T2DM_CAD groups with CAD group, metabolic hormones i.e., insulin, GIP and GLP-1 levels were significantly (fdr corrected p-value < 0.10) increased where IP-10 levels were decreased in T2DM_CAD as compared with CAD group.

### Serum protein marker levels in T2DM and CAD group compared with control group

Our data also indicated that metabolic hormone i.e., GIP, cytokine i.e., GM-CSF and apolipoprotein i.e., Apo-AI levels were significantly (fdr corrected p-value < 0.10) decreased while lipocalin-2 was significantly increased in T2DM group as compared with control group (fdr corrected p-value < 0.10). Metabolic hormone such as ghrelin and adipokines i.e., resistin, PAI-I, adipsin, and lipocalin-2 levels were significantly (fdr corrected p-value < 0.10) increased in CAD group and similarly cytokines eotaxin, IP-10, PDGF-BB levels, and apolipoprotein such as Apo-CII levels were significantly (fdr corrected p-value < 0.10) increased in CAD group as compared with control group. Metabolic hormones i.e., GIP and leptin levels were significantly (fdr corrected p-value < 0.05) decreased in CAD group as compared to control group (Table 2).

### Network analysis

**Fig. 2:**
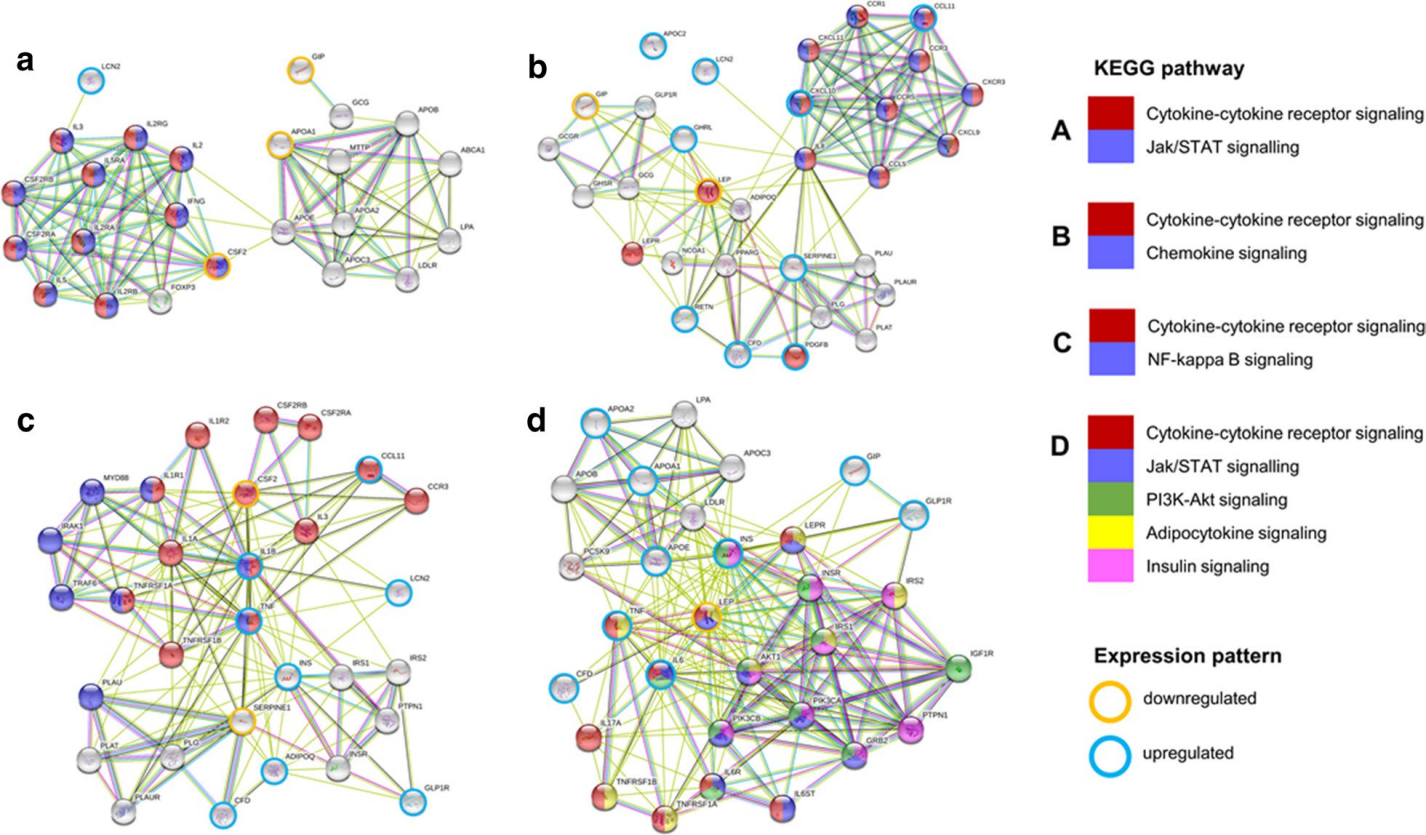
Subnetworks of the significant markers for T2DM, CAD and T2DM_CAD. **a** Network for the significant proteins in type 2 diabetes compared with control group. **b** Network for the significant proteins in CAD group compared with control group. **c** Network for the significant proteins in T2DM_CAD group compared with control group. **d** Network for the significant proteins in the T2DM_CAD group compared with T2DM group

Further, we conducted protein–protein interaction (PPI) network analysis using significantly (p ≤ 0.05) altered proteins in each disease using STRING 10.5 database. STRING database provides PPIs from experimental interactions from different sources combining text and data mining approaches. We constructed disease-specific PPI networks based on high confidence score threshold (STRING score ≥ 0.7). The Kyoto Encyclopedia of Genes and Genomes (KEGG) database was used to assign related gene categories into their associated pathways, through the STRING interface. KEGG pathway enrichment analysis was performed and results with multiple testing corrections were used for further analysis. False discovery rate (FDR) threshold ≤ 1% was applied. KEGG pathway analysis sheet was submitted as Additional file 1. The important processes were colored using the STRING analysis tool tab. The networks were downloaded and edited to highlight the upregulated and downregulated proteins. In T2DM group, three proteins were down-regulated and one protein lipocalin-2 was up-regulated. PPI network showed that these molecules were involved in the cytokine–cytokine receptor signaling and Jak/Stat signaling (Fig. 2a). Similarly, in CAD group, lipocalin 2, ghrelin, PAI-I (serpine1), adipsin (CFD), resistin, PDGF-BB, CCL11, IP-10 and APO-CII were upregulated, and GIP and leptin were down regulated. The network revealed that these molecules were closely associated with cytokine–cytokine receptor signaling and chemokine signaling (Fig. 2b). T2DM_CAD group GM-CSF (CSF2) and PAI-I levels were down regulated and TNF-alpha, IL-1β, CCL-11, lipocalin 2, insulin, GLP-1, adiponectin and adipsin were upregulated. All these proteins are involved in the cytokine–cytokine receptor signaling, NF-kB signaling, insulin signaling and adipocytokine signaling (Fig. 2c). In the T2DM_CAD category, nine markers GLP-1, GIP, Insulin, IL-6, Apo E, Apo-AI, Apo-AII, TNF-α, IL-6, and adipsin were upregulated and leptin was down regulated. These proteins were involved in the cytokine–cytokine receptor signaling, Jak-Stat signaling, PI3K-Akt signaling, adipocytokine signaling and insulin signaling when compared with T2DM (Fig. 2d).

### Correlation among protein markers

**Fig. 3:**
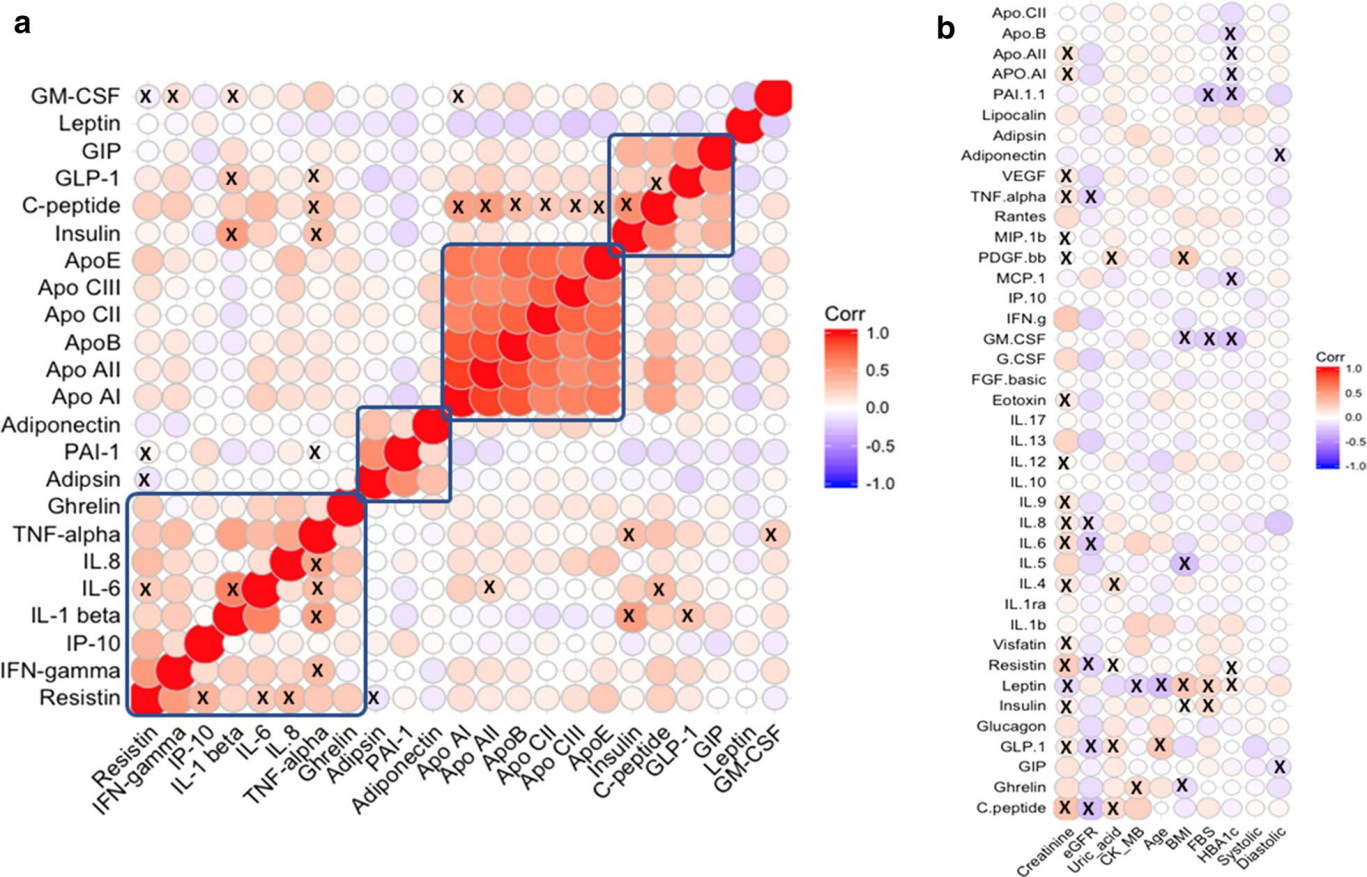
Heatmap showing **a** the mutual spearman correlations among the markers and **b** spearman correlations between the clinical characteristics on the horizontal axis and the markers on the vertical axes. Background colour indicates strength of association. R value 0.3 was set as threshold and significance was considered as p < 0.05

The correlation between protein markers were shown in Fig. 3a. Our correlation analysis between protein markers found that pro-inflammatory marker IL-6 positively correlated with IL-1β and TNF-α. Pro-inflammatory cytokine i.e., IL-8 is positively correlated with TNF-α. Adipokine like resistin is positively correlated with pro-inflammatory cytokines IP-10, IL-6, IL-8 and negatively correlated with the adipokine i.e., adipsin. Pro-inflammatory marker IL-1β positively correlated with metabolic hormones insulin, GLP-1 and pro-inflammatory marker TNF-α (Fig. 3a). GM-CSF is negatively correlated with the resistin and positively correlated with the IFN-Gamma, IL-1β, and Apo-AI. C-peptide levels were positively correlated with insulin and apolipoproteins (Apo-AI, Apo-E, Apo-B, Apo-AII, Apo-CII, Apo-CIII).

### Correlation among protein markers and clinical characteristics

The correlations between protein markers and clinical characteristics were shown in Fig. 3b. Our correlation analysis showed that fasting blood glucose levels were positively correlated with metabolic hormones insulin and leptin and negatively correlated with PAI-I and GM-CSF. Another important diabetic parameter HbA1c is positively correlated with leptin and resistin, and negatively correlated with cytokines i.e., GM-CSF, MCP-1, PAI-I and apolipoproteins i.e., Apo-AI, Apo-AII, and Apo-B. Diastolic blood pressure negatively correlated with GIP and adiponectin. However, systolic blood pressure not correlated with any of the protein markers. Renal marker creatinine positively correlated with C-peptide, insulin, GLP-1, resistin, visfatin, IL-4, IL-6, IL-8, IL-9, IL-12, eotaxin, TNF-α, VEGF, Apo-AI and Apo-AII and negatively correlated with leptin, PDGF-BB and MIP-1β. Estimated glomerular filtration rate (eGFR) is another factor which is used for the measurement of renal function is negatively correlated with metabolic hormones C-peptide, GLP-1, resistin, and pro-inflammatory markers, i.e. TNF-α, IL-6 and IL-8. Uric acid positively correlated with C-peptide, GLP-1, IL-4, and PDGF-BB and negatively correlated with resistin. CK-MB, a biomarker for the myocardial infarction, is positively correlated with ghrelin and negatively correlated with leptin. Age positively correlated with GLP-1 and negatively correlated with leptin. BMI is positively correlated with leptin and PDGF-BB, and negatively correlated with metabolic hormones i.e., ghrelin, insulin and cytokines i.e., IL-5 and GM-CSF.

### Machine learning classification methods for characterizing the disease groups

**Fig. 4:**
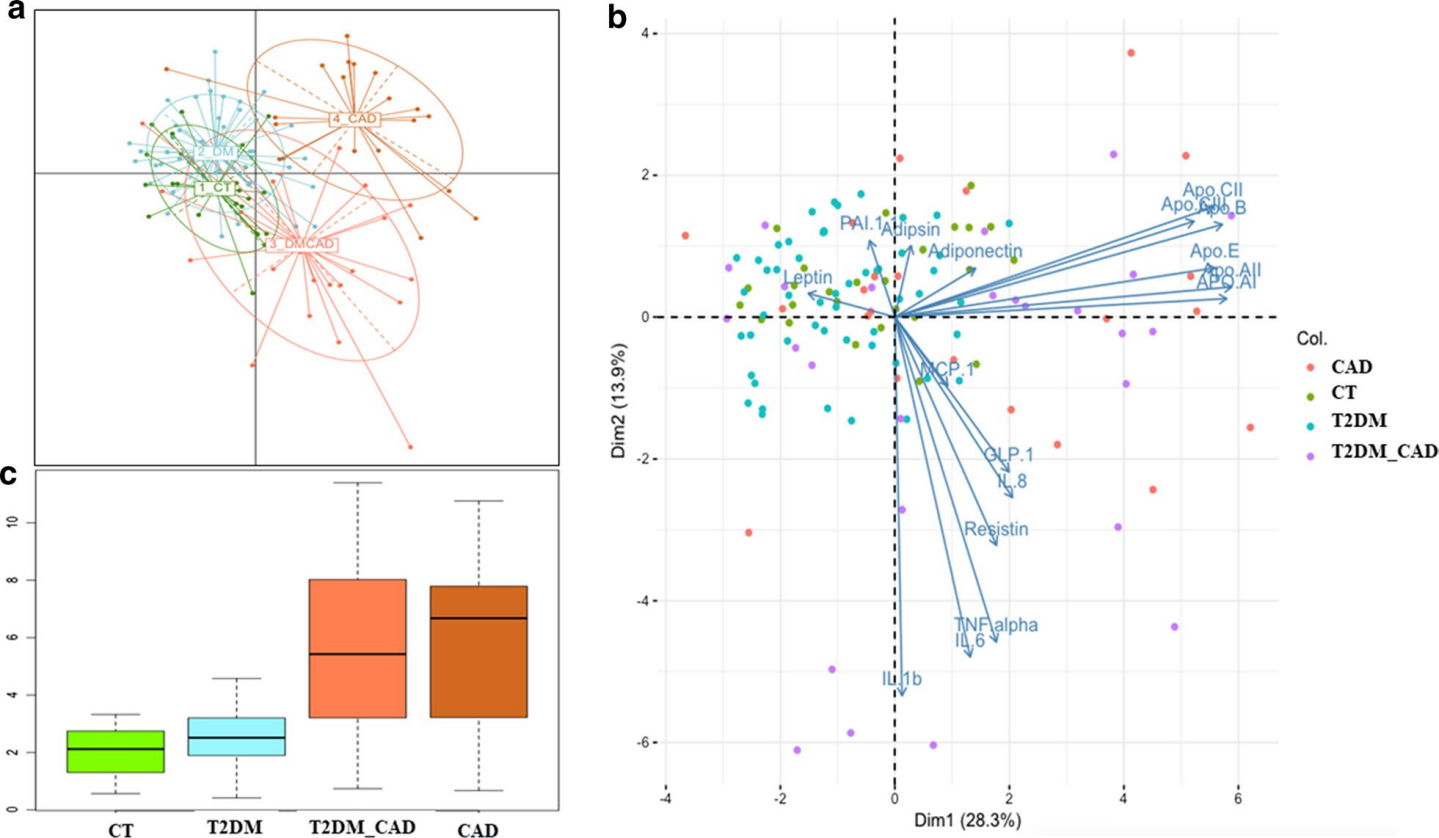
**a** Between class analysis (BCA) ordination plot representing marker profiles of the different subjects. Subjects belonging to different groups are coloured differently (as indicated) and connected with the centroid profiles of each group. Between class analysis reveals distinct biomarker profiles for T2DM_CAD and CAD. However, T2DM group of subjects are observed to have marker profiles, relatively similar to that of controls. **b** Principal Component Analysis (PCA) plot representing marker profiles of the different subjects and **c** boxplot of the within group marker profile variations (computed using J-Divergence measures) further reveals that there is a significant high degree of variability in the marker profiles in both CAD and T2DM_CAD groups (as compared to the control and T2DM groups)

We used two supervised analysis methods to distinct disease groups based on the protein markers i.e., Between Class Analysis (BCA) and Principal Component Analysis (PCA). BCA performed between class analysis to distinct the diseases groups with marker profiles. Our BCA analysis revealed that distinct biomarkers profile for the T2DM_CAD and CAD groups. However, T2DM group has marker profiles, relatively similar to that of control group. BCA ordination plot shown in Fig. 4a. Subjects belonging to different groups are colored differently (as indicated in the figure legend) and connected with the centroid profiles of each group.

Furthermore, principal component analysis (PCA) of the serum markers showed decent separation of samples from patients with CAD and T2DM_CAD from both controls and T2DM individuals based on the most decisive component of the dataset (Fig. 4b). Dimension 1 (Dim 1) of the PCA accounted for 28.3% variation, while Dimension (Dim 2) accounted for 13.9% variability. Further, we have presented within the group marker profile variations, which revealed that there is a high degree of variability in the markers within CAD and T2DM_CAD as compared to the control and T2DM group (Fig. 4c).

**Fig. 5:**
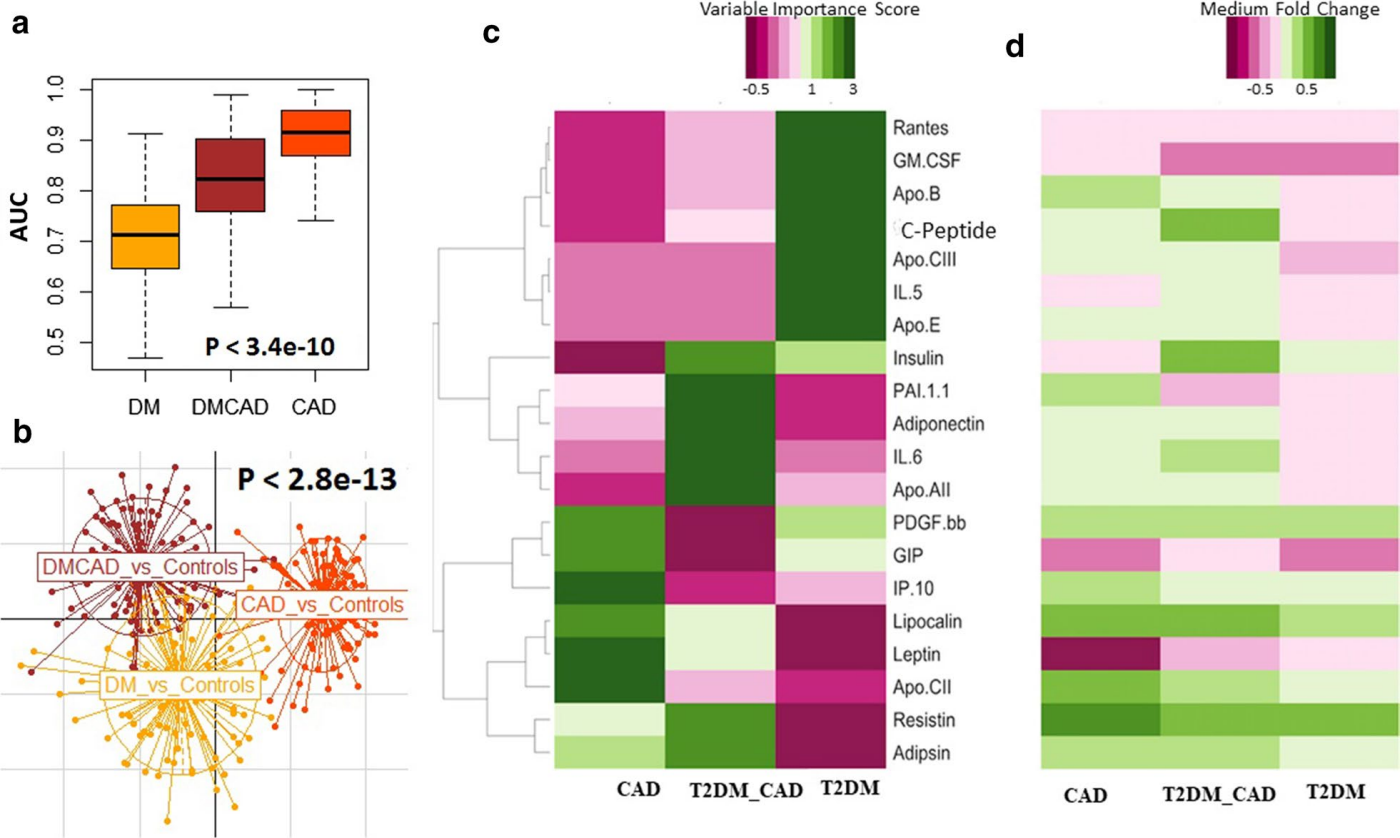
**a** Classification Area under the Curves (AUCs) of Random Forest-based classifiers (trained on the marker profiles) for predicting the different disease classes with respect to healthy controls. For each disease state, classification accuracies were obtained after 100 iterations, where in each iteration, the model was trained on 50% of the data and validated/tested on the rest 50%. **b** PCA plots of the vectors of the ranked feature importance scores for each iterations for three diseases (300 vectors for 100 iterations for each of the three diseases), showing significantly distinct profiles of the feature importance for classification of the three diseases (PERMANOVA p-value < 2.8e−13). **c** Variable importance scores of the markers identified to be optimal for at least one of the three comparisons (CT v/s T2DM, CT v/s T2DM_CAD and CT v/s CAD). **d** Fold change of the median abundances of the corresponding markers for each disease state versus the controls

For each disease groups among control prediction (Fig. 5), we performed 100 iterations, where in each iteration; we trained a random forest classification model on 50% of the dataset (based on the protein profiles), and tested it on the rest 50% (2-fold cross validation; rather than the 90% to 10% training: testing ratio, thereby reducing the over-fitting aspect). In other words, each iteration involved the random forest classification trained on a different subset of control and diseased samples, and tested on a completely non-overlapping set. The two cross validation addresses that the models do not over-fit. Furthermore, the 100 iterations gave us the statistical power to explore the entire landscape of individuals (available in the current study) to judge the power of each classification strategy. The classification power of each feature was then computed as the mean feature rank across all the 100 iterations. Our random forest classifier approach is given predictive models to distinct the different diseases like T2DM, CAD, and T2DM_CAD respect to control group. We also found the random forest classifier to predict T2DM_CAD with respect to T2DM. Accuracy, variable importance score and median abundance of the markers for each disease states were shown in Fig. 5a. The iterative approach gave us the statistical power to not only compare the classification efficiency for the three diseases using the protein profiles (p < 3.4e−10 using Kruskal–Wallis H-test, CAD > T2DM_CAD > DM), but also validated the stability of the features for prediction across an entire landscape of subjects (Fig. 5b). Principal Coordinate Analysis for the 100 ranked feature importance profiles (obtained in each iteration) for each disease, indicates that the feature importance profiles are relatively similar to each other for a given disease and significantly from those of other diseases (PERMANOVA p < 2.8e−13).

Briefly, Random Forest (RF) classifier classified T2DM group with respect to the control group by nine markers (IL-1β, GM-CSF, glucagon, PAI-I, rantes, IP-10, resistin, GIP, Apo-B; accuracy 76%, sensitivity 72%, specificity 81%, AUC 0.72), CAD was predicted with respect to the control by 14 markers (resistin, PDGF-BB, PAI-1, lipocalin-2, leptin, IL-13, eotaxin, GM-CSF, Apo-E, ghrelin, adipsin, GIP, Apo-CII, IP-10; accuracy 86%, sensitivity 85%, specificity 87.5%, AUC 0.84); T2DM_CAD was predicted by 12 markers (insulin, resistin, PAI-1, adiponectin, lipocalin-2, GM-CSF, adipsin, leptin, Apo-AII, rantes, IL-6, and ghrelin; accuracy 92%, sensitivity 92.3%, specificity 90%, AUC 0.92); (Fig. 5c). T2DM_CAD was also classified well with respect to T2DM by nine markers (adiponectin, C-peptide, resistin, IL-1β, ghrelin, lipocalin-2, Apo-AII, IP-10, Apo-B; accuracy 85.7%, sensitivity 86.9%, specificity 78.5%, AUC 0.76) (6a–c). These all classifiers were considered as significant p < 0.05 and shown in Table 3.

**Fig. 6:**
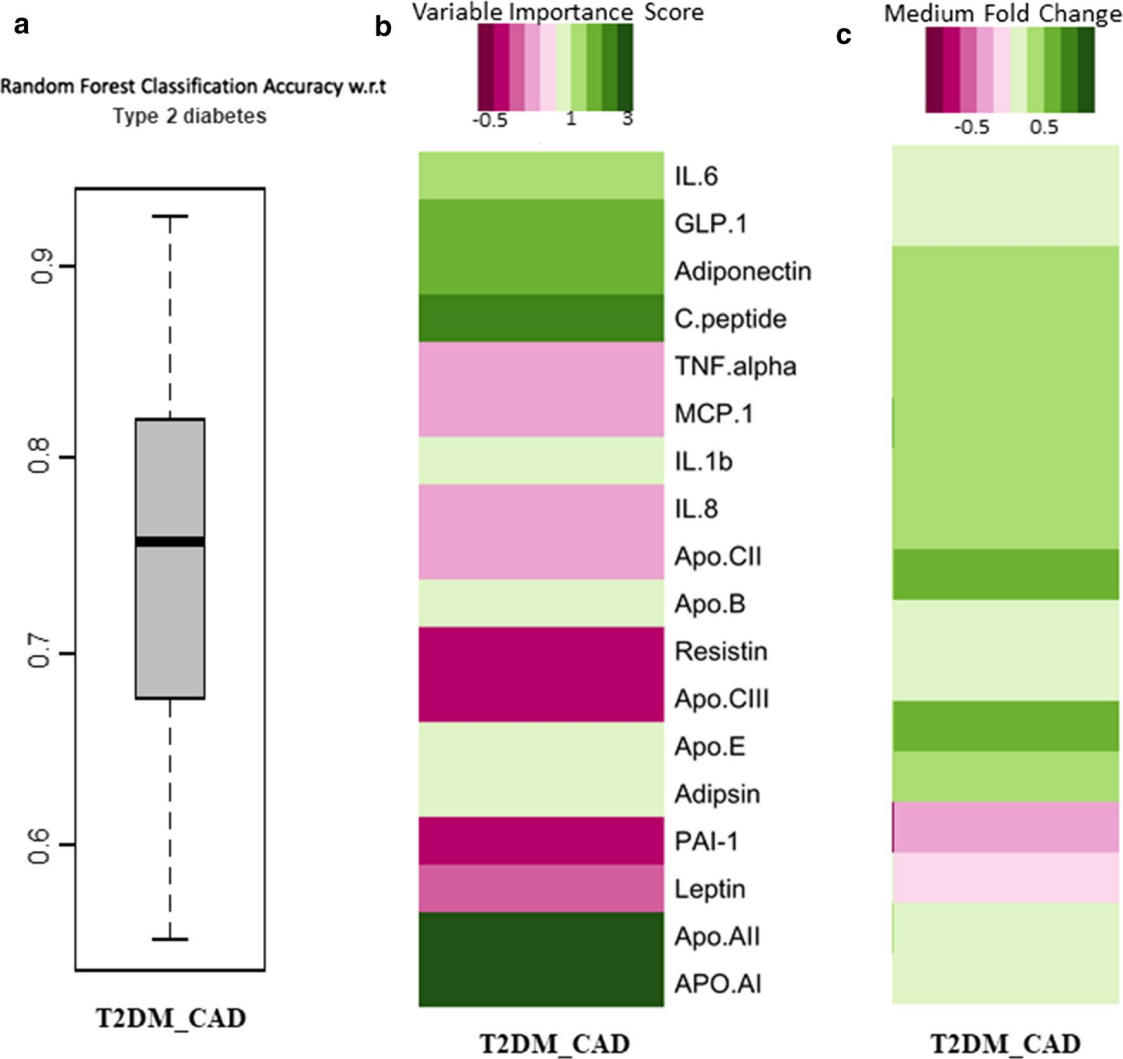
**a** Classification area under the curves (AUCs) of random forest-based classifiers (trained on the marker profiles) for predicting the T2DM_CAD with respect to T2DM. For each disease state, classification accuracies were obtained after 100 iterations, where in each iteration, the model was trained on 50% of the data and validated/tested on the rest 50%. **b** Variable importance scores of the markers identified to be optimal for at least one of the three comparisons (T2DM v/s T2DM_CAD). **c** Fold change of the median abundances of the corresponding markers for each comparison (T2DM v/s T2DM_CAD)

**Table 3:** Classification performance of marker profile based on random-forest classifiers for different pairs of groups

	Optimal markers giving the separation more than > 1 VIS	Classification accuracy (%)	Sensitivity (%)	Specificity (%)	AUC (Median)	p value
Control vs T2DM	Total 9 markers i.e., IL-1beta, GM-CSF, glucagon, PAI-I, rantes, IP-10, resistin, GIP, Apo-B.	76	72	81	0.72	< 0.0009
Control vs CAD	Total 14 markers i.e., resistin, PDGF-BB, PAI-1, lipocalin-2, leptin, IL-13, eotaxin, GM-CSF, Apo-E, ghrelin, adipsin, GIP, Apo-CII, IP-10.	86	85	87.5	0.84	3.5e−6
Control vs T2DM_CAD	Total 12 markers i.e., insulin, resistin, PAI-1, adiponectin, lipocalin-2 GM-CSF, adipsin, leptin, apo-AII, rantes, IL-6, Ghrelin.	92	92.3	90	0.92	4.2e−10
T2DM vs T2DM_CAD	Total 9 markers i.e., adiponectin, C-peptide, resistin, IL-1beta, Ghrelin, lipocalin-2, Apo-AII, IP-10, Apo-B	85.7	86.9	78.5	0.76	4.3e−6

For each pair of groups, the Random Forest classifications were obtained with 10-fold cross validation (there were 1000 iterations where in each iteration the classifiers were trained on 90% of the subjects, while the rest 10% were used for prediction). Top discriminatory marker features for each pair wise classification. Fisher’s exact test were then performed on the confusion matrix, in order to judge the significance of the prediction profile

*VIS* variable importance score, *AUC* area under curve, AUC is mentioned as median

## Discussion

In the process of development of CAD in T2DM, multiple pathophysiologic processes including hyperglycemia, hyperinsulinemia, insulin resistance, dyslipidemia, chronic low-grade inflammation, oxidative stress, endothelial dysfunction, vascular calcification, and hypercoagulability were involved [17]. However, these all mechanisms were responsible together to alter the plasma levels of metabolic hormones, inflammatory mediators, adipokines and apolipoproteins in diabetes and associated cardiovascular disease complications [18]. These individual mechanisms and their interplay were not fully understood in the diabetic disease progression. In the present study, we were interested to find the simultaneous induction of various protein markers in diabetes and CAD based on the diseases mechanisms related to metabolic hormonal regulation, inflammation, adipogenesis, and atherogenesis. Our interest was to characterise T2DM by a panel of protein markers that accelerates CAD progression in Indian subjects. Identification of specific markers in Indian T2DM patients is very important considering the high prevalence of type 2 diabetes and its complications specially CAD in India.

The present study showed that metabolic hormones like GLP-1, C-peptide and insulin levels were significantly increased in T2DM_CAD as compared with control. Similarly, GLP-1 and C-peptide levels were significantly increased in TDM_CAD as compared with T2DM. Previously, it was reported that increased insulin and C-peptide levels were independently associated with increased risk of coronary artery disease in T2DM subjects. Interestingly, hyperinsulinemia accelerates atherosclerosis in diabetic patients than non-diabetic patients [19, 20]. In contrast, GLP-1 other than metabolic regulation shows anti-atherosclerotic effect [21]. Increased GLP-1 levels in T2DM_CAD group that we observed in the present study might be the compensatory response to the atherosclerotic effect. In the present study leptin level were significantly lower in CAD patients but increased when diabetes is associated with CAD. Al-Daghri et al. reported the association of increased leptin levels with severity of both metabolic syndrome and diabetes associated with coronary heart diseases [22]. Many clinical reports suggest that leptin might play a key link between metabolism and inflammation, across different age categories, ranging from pediatric to geriatric patients with diabetes or other cardiovascular risk factors [23]. Further, leptin is able to induce production of C-reactive protein by endothelial cells [24]. The increase local availability of leptin in the vascular wall can in turn exert pro-atherothrombotic effects both on endothelial cells and smooth muscle cells [25, 26].

Among all pro-inflammatory cytokines i.e., TNF-α and IL-1β levels were increased in T2DM_CAD subjects as compared with control subjects. As per the previous literature, TNF-α induces insulin resistance by inhibiting IRS-1 phosphorylation and GLUT-4 expression, and elevated in patients with heart failure, and myocardial ischemia reperfusion [27]. Pu et al. reported the association of increased TNF-α levels with CAD among T2DM patients [28]. Increased TNF-α and IL-1β levels along with hyperglycemia helps to develop atherosclerosis [29, 30]. Similarly, another pro-inflammatory marker IL-6 increased in both CAD and T2DM_CAD groups. However, the significant increase was observed in T2DM_CAD group as compared with T2DM. Increased IL-6 levels disturb the glucose metabolism and contribute to the development of insulin resistance. It is also reported earlier that IL-6 levels were positively correlated with CAD [31]. Present study showed that pro-inflammatory markers i.e., TNF-α, IL-1β, and IL-6 together induce chronic inflammation condition to promote CAD in diabetes. Recently, CANTOS study reported that treatment with canakinumab, a monoclonal antibody targeted against IL-1beta in myocardial infraction patients showed dose-dependent decrease in IL-6 levels [6]. Serum markers like PDGF-BB, IP-10, resistin and PAI-1 levels were significantly increased in the CAD group, therefore representing specific markers for the CAD in the absence of diabetes. All these parameters help to develop atherosclerosis as reported earlier [32–37].

In our study, GM-CSF levels decreased in T2DM and T2DM_CAD group as compared with control group. GM-CSF induces activation of monocytes/macrophages and mediates differentiation to other states that participate in immune responses. Previously, researchers reported that GM-CSF protects from diabetes by increasing a tolerogenic dendritic cells population [38]. Recently, Al-Hassnawi et al. reported decreased GM-CSF levels in type 2 diabetes patients and found indirect association with blood glucose levels [39]. Present study also confirmed that GM-CSF could be used in the disease progressive marker for diabetes and diabetes with CAD. Similarly, eotaxin levels were significantly increased in CAD and T2DM_CAD group. Researchers previously reported that increased eotaxin levels were associated with CAD and coronary atherosclerosis [40]. Therefore, elevation of eotaxin levels might be more important for atherosclerosis development in diabetes subjects. Increased adipsin levels were observed in CAD and T2DM_CAD group while lipocolin-2 levels were increased in T2DM, CAD and T2DM_CAD groups. Researchers reported that adipsin promotes lipid accumulation and adipocyte differentiation, and improves beta cell function [41, 42]. In previous literature, it was reported that increased lipocolin-2 in serum is positively correlated with insulin resistance and inflammation in T2DM patients [43, 44].

The association of Apo-AI, Apo-AII and Apo-CII levels was reported with atherosclerotic occlusive disease, CAD and type 2 diabetes associated with CAD [45–47]. In the present study, Apo-AI, Apo-AII and Apo-CII levels were increased in CAD and T2DM_CAD group, however, significantly increased was observed only in CAD group. As per the previous literature increased Apo-AI shows cardio-protective effect and thus improvement of Apo-AI expression is considered as a potential therapeutic strategy to inhibit atheroma formation [3]. However, ApoA-I Milano product (MDCO-216) and wild-type ApoA-I product (CER-001) failed to promote regression of coronary atherosclerosis compared with placebo. Further another ApoA-I product, CSL112, recently entered a phase III cardiovascular outcomes trial [48]. Hope this study may come up with a fruitful result. Researchers reported that Apo-AII promotes insulin resistance and disturbs body fat homeostasis [49]. Increased Apo-AII levels promote development of atherosclerosis by disturbing the reverse cholesterol transport and antioxidant properties of HDL [49–53]. Similarly, Apo-CII plays an important role in triglyceride rich lipoprotein metabolism, and positively correlates with increased CAD and coronary heart diseases (CHD) [54].

Between class analysis (BCA) was performed to distinct the disease groups with marker profiles. Our BCA analysis revealed that distinct biomarkers profile was observed for the T2DM_CAD and CAD groups but no difference between control and T2DM group. Thus the distinct biomarker profile between groups is depends on high degree of glycaemia, duration of diabetes state and its complications. Principal component analysis (PCA) of the serum markers showed decent separation of samples with high degree variability with CAD and T2DM_CAD from controls and type 2 diabetes based on the most decisive component of the dataset.

**Fig. 7:**
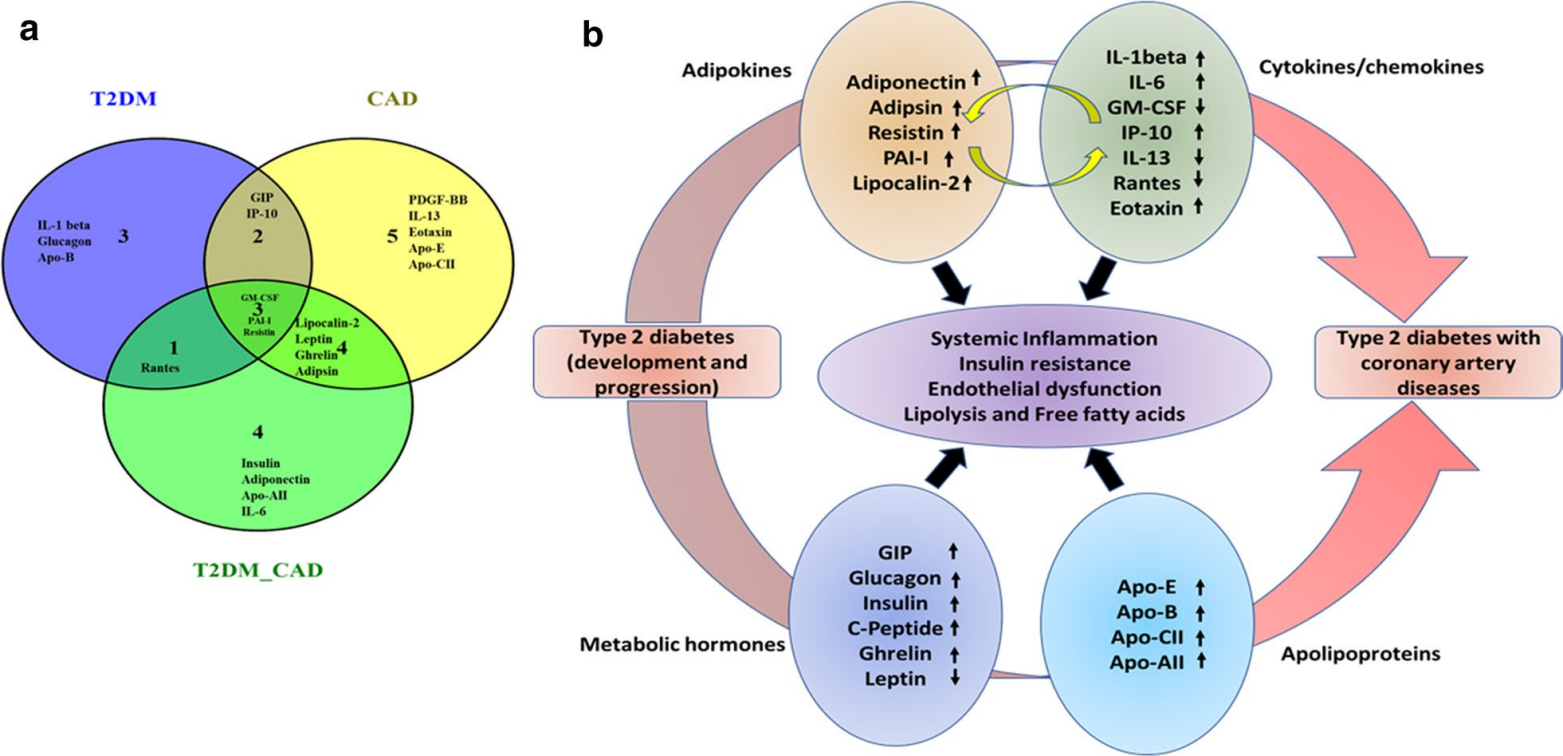
**a** Venny diagram represented common and unique protein markers from the RF classifier to distinct type 2 diabetes, CAD, and T2DM_CAD as compared with control. **b** Protein markers that responsible for development and progression of diabetes and associated coronary artery disease complication. Different pathological protein markers i.e., adipokines, cytokines, metabolic hormones and apolipoproteins (markers which were classified in RF classifier Table 3) may act as mediators in the initiation of insulin resistance, systemic inflammation, endothelial dysfunction and increase lipolysis and free fatty acids. Up arrow resembles upregulated proteins and down arrow resembles downregulated markers

We have also analysed our data using random forest classifier approach, which is a predictive model to distinct the different diseases like T2DM, CAD, and T2DM_CAD respect to control group, and classified T2DM_CAD group in respect to T2DM group. All these protein markers from random forest (RF) classifier were further used to made venny diagram to represent common and individual markers to distinct each disease group from other group (Fig. 7a). We found that GM-CSF, PAI-I and resistin were common classifiers for the T2DM, CAD, T2DM_CAD diseases. While IL-1β, glucagon and Apo-B are individual markers for type 2 diabetes, PDGF-BB, IL-13, eotaxin, Apo-E, and Apo-CII are the individual markers for the CAD group. Only four markers like insulin, adiponectin, Apo-AII and IL-6 are the individual markers for T2DM_CAD group. Six serum markers representing metabolic hormones (leptin, ghrelin) and adipokines (resistin, adipsin, PAI-1, lipocalin-2) are common between CAD and T2DM_CAD group while serum markers from inflammation, cytokines and apolipoproteins observed in both groups are completely different. Similarly, four serum markers representing inflammation (GM-CSF), adipokines (resistin, PAI-1) and apolipoproteins (Apo B) are common between T2DM and T2DM_CAD groups while serum markers from metabolic hormones observed in both groups are completely different. The classifier analysis showed that few plasma markers from T2DM_CAD group were common with T2DM and CAD marker panels. These common plasma markers confirm the involvement of common pathologies among T2DM, CAD and T2DM_CAD.

Few plasma protein markers i.e., rantes, IL-13, glucagon and Apo B were picked up with the absence of statistically significant differences. Rantes (CCL5) an inflammatory marker, secreted by adipocyte and contributes to leukocyte infiltration. Previous researcher reported that increased levels of circulatory rantes observed in obesity, impaired glucose tolerance, type 2 diabetes, and coronary artery diseases [55, 56]. In contrast, Podolec et al. reported that severe coronary atherosclerosis correlated with decreased rantes levels [57]. Our data suggested that rantes could be a marker for type 2 diabetes and type 2 diabetes with CAD. Similarly, anti-inflammatory molecule IL-13 is a classifier of CAD panel without any significant change. Decreased serum IL-13 levels in T2DM subjects play a role in impaired glucose uptake and metabolism [58]. Similarly, another study showed decreased IL-13 levels in patients with coronary artery disease subjects [59]. Metabolic hormone glucagon has emerged as one of the marker for the type 2 diabetes classifier; however, there was no significant difference. Metabolic hormones glucagon and insulin together regulate glucose production by stimulatory and inhibitory actions, respectively [60]. In our study, decreased glucagon levels, and increased insulin levels were observed in type 2 diabetes group. However, in T2DM_CAD group, both the levels were increased which shows impairment of the glucose regulation. Apolipoproteins like Apo-B and Apo-E were classifiers for the T2DM and CAD, respectively and Apo-B, was a classifier marker for the T2DM_CAD. It is well known that Apo-B and Apo-E were risk factor for coronary artery diseases and cardiovascular mortality [61]. Researchers also reported that Apo-B is associated with the incident type 2 diabetes and better predictor for coronary artery diseases among diabetic patients [62, 63]. As reported earlier our study also classified Apo-B a marker for T2DM and T2DM_CAD when compare to control and T2DM, respectively.

Furthermore, all these significant protein markers in study groups compared with control group and T2DM_CAD group compared with T2DM were analyzed using STRING database to find the possible cellular signaling pathways (Fig. 2). Circulatory protein markers with significant change in respect to control or T2DM were used to build a closely associated network of known pathways. Network analysis revealed the complexity of T2DM_CAD that linked to several inflammatory and insulin resistance pathways like cytokine receptor, Jak-STAT, PI3K-Akt, adipocytokine and insulin signaling pathways (Fig. 2d). To understand the overall affected pathways throughout the disease progression starting from healthy to diabetes-to-diabetes with CAD, we combined all significant circulatory markers to link with the crucial cellular proteins that could be affected during the disease progression (Fig. 2b). PTPN1 found from the network study (Fig. 2c) is linked to the common network pathway and modulates insulin resistance [64] through Jak-Stat, insulin receptor substrates (IRS1 and IRS2) and leptin signaling pathway [64]. All these proteins are already being explored as targets for the treatment of diabetes in diverse studies. Hyperinsulinemia that observed in T2DM and T2DM_CAD groups, may down regulate IRS1 and IRS2 via p38-MAPK and triggers insulin resistance in liver and skeletal muscle. Alteration of leptin found in T2DM_CAD also effects phosphatidylinositol 3-kinase-Akt (PI3K-Akt) signaling pathway via Jak2 activation of IRS1 and IRS2 (Fig. 2d). Together these proteins contribute to the dysregulation of glucose and lipid metabolism, mitochondrial biogenesis, calcium-handling, fibrosis, and motor gene expression, culminating in cardiovascular complications. Pro-inflammatory marker IL-6 signaling via IL6R and STAT3 is a contributor for the vascular inflammation in vessel wall or fatty streak. Another pro-inflammatory marker i.e., IL-1β that belongs to IL-1 family, binds with the IL-1R type I (IL-1RI) and induces a downstream signal via numerous inflammatory kinases, such as Myd88, ERK, JNK and NF-κB leading to transcription of several inflammatory genes like cytokines and chemokines (Fig. 2c). Some signaling molecules specially, NF-kB also overlap with toll like receptors (TLRs) signaling [65]. Recently, the transcription factor high-mobility-group AT-hook 1 (HMGA1) has been linked to NF-kB activation, and involved in inflammation and in the pathogenesis of insulin resistance. It has been demonstrated that HMGA1 is associated to both the risk for diabetes and the risk of developing cardiovascular complications [66].

With the help of the network analysis, we were able to identify several cellular network proteins like PTPN1, AKT1, INSR, LEPR, IRS1, IRS2, AKT1, IL1R2, IL6R, PCSK9 and MYD88, which are responsible for regulating inflammation, insulin resistance, and atherosclerosis. Several adipokines like resistin, adiponectin, lipokolin-2 and IL-6 contribute to the development of insulin resistance, type 2 diabetes and cardiovascular diseases. We also found that apolipoproteins and cytokines were tightly connected with each other in the network and contribute to the development as well as progression of diabetes and diabetes with coronary artery diseases. Using this information, we summarized this into a model of disease progression (Fig. 7b) where systemic inflammation, insulin resistance, endothelial dysfunction, lipolysis and free fatty acid pathways come together in the disease pathogenesis.

## Conclusion

In conclusion, we have identified protein marker profile for diabetes and diabetes associated with CAD in Indian patients. Using nonparametric-based machine learning approach, we have classified each disease by a set of distinct protein markers. These multiple marker classifiers may be useful to find the diseases progression and monitor treatment. However, the major limitation of the present study is the cross-sectional nature of the study with small sample size and did not confirm the causality. Further, these analyses are in majority exploratory and need to confirm the findings in a separate cohort with a large number of samples from Indian populations.

## Additional file

**Additional file 1.** KEGG pathway analysis sheet.

## Abbreviations

T2DM: type 2 diabetes
CAD: coronary artery diseases
IL: interleukin
IL-1ra: IL-1 receptor antagonist
IFN-gama: interferon (IFN)-gamma
IP-10: IFN-gamma inducible protein
MCP-1: monocyte chemotactic protein
MIP-1α: macrophage inflammatory protein
PDGF-BB: platelet-derived growth factor
TNF-α: tumor necrosis factor (TNF)-alpha
VEGF: vascular endothelial growth factor
PAI-1: plasminogen activating factor
APO: apolipoprotein
